# Inversion of Left Atrial Appendage Will Cause Compressive Stresses in the Tissue: Simulation Study of Potential Therapy

**DOI:** 10.3390/jpm12060883

**Published:** 2022-05-27

**Authors:** Salvatore Pasta, Julius M. Guccione, Ghassan S. Kassab

**Affiliations:** 1 Department of Engineering, Viale delle Scienze, Università degli Studi di Palermo, 90128 Palermo, Italy; salvatore.pasta@unipa.it; 2 Department of Surgery, University of California San Francisco, San Francisco, CA 94143, USA; julius.guccione@ucsf.edu; 3 California Medical Innovations Institute, 11107 Roselle, San Diego, CA 92121, USA

**Keywords:** finite element method, atrial fibrillation, atrophy, fibrosis

## Abstract

In atrial fibrillation (AF), thromboembolic events can result from the particular conformation of the left atrial appendage (LAA) bearing increased clot formation and accumulation. Current therapies to reduce the risk of adverse events rely on surgical exclusion or percutaneous occlusion, each of which has drawbacks limiting application and efficacy. We sought to quantify the hemodynamic and structural loads of a novel potential procedure to partially invert the “dead” LAA space to eliminate the auricle apex where clots develop. A realistic left atrial geometry was first achieved from the heart anatomy of the Living Heart Human Model (LHHM) and then the left atrial appendage inversion (LAAI) was simulated by finite-element analysis. The LAAI procedure was simulated by pulling the elements at the LAA tip and prescribing a displacement motion along a predefined path. The deformed configuration was then used to develop a computational flow analysis of LAAI. Results demonstrated that the inverted LAA wall undergoes a change in the stress distribution from tensile to compressive in the inverted appendage, and this can lead to resorption of the LAA tissue as per a reduced stress/resorption relationship. Computational flow analyses highlighted a slightly nested low-flow velocity pattern for the inverted LAA with minimal differences from that of a model without inversion of the LAA apex. Our study revealed important insights into the biomechanics of LAAI and demonstrated the inversion of the stress field (from tensile to compressive), which &can ultimately lead the long-term resorption of the LAA.

## 1. Introduction

Atrial fibrillation (AF), the most common sustained arrhythmia, affects 3–6 million Americans and increases the risk of stroke by 4 to 6 times on average [1,2]. AF prevalence and disease burden increase with age, accounting for 15% of all strokes, and with greater associated morbidity and mortality than non-AF related strokes [3]. In people >80 years old, AF is the direct cause of 1 in 4 strokes [1]. AF and related disorders have high individual and societal costs, ~$26 B per year in the US [4], and incidence is projected to more than double by 2035 [5].

The left atrial appendage (LAA) extends from the LA and creates a side chamber, which can be a site of increased clot formation and accumulation in AF. The LAA in a low-flow state as in AF is the nidus for >90% of thrombus formation [6,7], where the rapid contraction of the heart that accompanies AF can initiate the release of emboli and the consequent risk of stroke. Although the risk of thromboembolic events is reduced with long-term oral anticoagulation therapy, it is contraindicated in 7.8% of newly diagnosed AF patients [8], and only 50–60% of eligible patients with AF receive it [9]. Percutaneous [10,11,12] and surgical strategies [13,14] to occlude or exclude the LAA have been developed to reduce the risk of thromboembolic events, but current devices have major complications (e.g., perforation, migration, and incomplete closure) and disadvantages (e.g., high-cost and foreign body retention). Compared to endocardial occlusion, epicardial clipping or exclusion seems to have better hemodynamic and neurohormonal effects, but it is technically more difficult to achieve [12,15,16].

**Figure 1:**
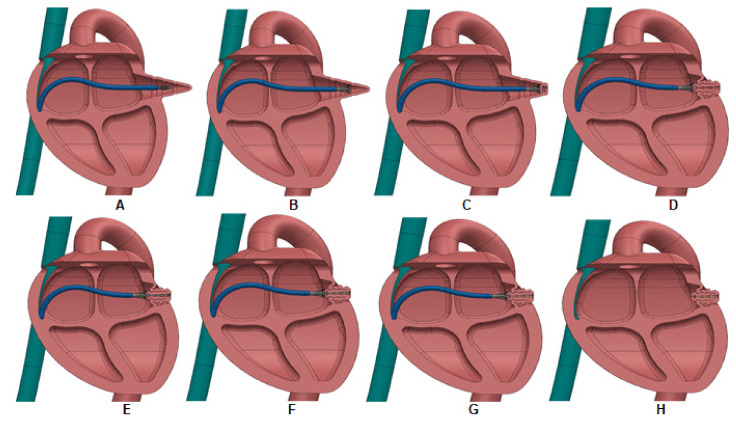
Schematic of LAA closure device and approach. The progression of the procedure is from (**A**–**H**). (**A**): Insert device into the LAA. (**B**): Deploy suction flute. (**C**): Suction onto inside distal wall of the LAA. (**D**): Pull the LAA inward and invert it. (**E**): Extend needle into inverted space. (**F**): Inject adhesive through needle-tip hypo-tube wire with side holes (blind closed tip). (**G**): Retract needle and maintain suction until adhesive cures. (**H**): Remove closure device leaving only tissue glue behind. The inversion of the LAA apex is not to scale, and can be much less, depending on the length of the LAA.

In this study, the feasibility of resorption of the “dead” LAA space without the need of a specific device left behind was investigated by computational modeling. Specifically, the procedure for the treatment of LAA uses a suction-based catheter device to latch the distal portion of the LAA endocardium (Figure 1), enabling partial LAA inversion (LAAI) as shown previously by our group [17]. The fundamental hypothesis is that the partial inversion of the LAA changes the stress distribution (from tensile to compressive) in the inverted appendage. An increase in tensile stress is known to cause tissue growth (e.g., hypertrophy in hypertension), whereas a decrease will cause resorption (e.g., atrophy in hypotension) as per a stress-growth law [18]. To test this hypothesis, the LAAI procedure was simulated using the realistic and accurate heart anatomy of the Living Heart Human Model^®^ (LHHM) [19]. Then, the fluid flow circulating in the inverted appendage was assessed by computational-fluid dynamics and compared to that of LAA. Several structural and hemodynamic parameters were extrapolated to assess the feasibility of the LAAI procedure.

## 2. Materials and Methods

### 2.1. LAAI Structural Analysis

The geometry of the left atrium (LA) including the ear-shaped sac of the LAA was extrapolated from that of the LHHM representing the ideal average heart anatomy of a middle-aged healthy male. No human data was used in this study, and thus no authorization is needed by ethical committee. The LA geometry was then discretized with 33,632 triangular elements (S3) as a shell layer with uniform thickness of 2 mm [20]. A nearly incompressible material with an infinitesimal value of D and material density of ρ = 1.06 × 10^−9^ kg/mm^3^ was used. Moreover, the biomechanical behavior of the LA tissue wall was modeled as a hyper-elastic and isotropic material using the third-order Ogden’s strain energy function:

(1) $$W\;=\;\overset{3}{\underset{i=1}{\sum }}\frac{\mu_{i}}{\alpha_{i}}(\lambda_{1}^{\alpha_{i}}\;+\;\lambda_{2}^{\alpha_{i}}\;+\;\lambda_{3}^{\alpha_{i}}\;-\;3)$$

where $\lambda_{1}$ are the principle stretches with three pairs of material parameters μ and α. Specifically, material descriptors were $\mu_{1}$ = −56.13 MPa, $\alpha_{1}$= 8.65, $\mu_{2}$ = 42.88 MPa, $\alpha_{2}$ = 10.03,$\;\mu_{3}$ = 13.59 MPa and $\alpha_{1}$ = 6.82 as obtained from the fitting of the passive myocardium [20].

To model the LAAI procedure, we first simulated the catheter clamping of the LAA by constraining the element nodes at the distal apex of the LAA. Then, the inversion was simulated pulling the clamped elements inside the heart by prescribing a displacement motion along a predefined path (i.e., the LAA centerline). Specifically, the clamped nodes were coupled to the centerline end point of LAA while the motion was implemented though connector assignments of constrained nodes in ABAQUS commercial software (ABAQUS v2020, Dassault Systèmes, Waltham, MA, USA). For each time increment, the displacement was 0.03 mm and was kept upon LAA inversion. To account for blood pressure, a uniform pressure of 1.3 mmHg was applied to LA inner surface. For boundary conditions, the distal ends of the pulmonary veins were fixed in all directions. The general contact algorithm with frictionless condition was adopted to consider the interaction of LAA tissue wall with itself during the retraction process. Simulations were carried out using ABAQUS/Explicit solver to account for the non-linear problem with large deformation and complex contact conditions. Energy was monitored to ensure the ratio of kinetic energy to internal energy remained less than 10%, and a variable mass-scaling technique was adopted to keep the time step less than 10^−6^. Post-processing was carried out with Ensight software by overlaying the geometry of the whole LHHM heart.

### 2.2. LAAI Fluid Dynamic Analysis

Once simulation of LAAI was accomplished, the deformed configuration of the LAA was exported to generate the fluid domain and thus assess the left heart hemodynamic environment by unsteady computational flow analysis. Specifically, the inner volume of the LAA wall was meshed with 2,411,143 tetrahedral elements using the ICEM CFD (v21.0, ANSYS Inc., Canonsburg, PA, USA). Mesh quality check was evaluated by grid-convergence index analysis to quantify the discretization error using the pressure gradient on the LAA wall as convergence parameter, as previously [21]. The blood flow was assumed as laminar and incompressible with non-Newtonian viscosity described by the Carreau model [22]. The Navier–Stokes equations governing fluid motion were solved with an implicit algorithm in FLUENT (v21, ANSYS Inc., Canonsburg, PA, USA), which has been previously used to resolve high-frequency, time-dependent flow instabilities encountered in complex cardiovascular anatomies [23,24]. Pressure-implicit with splitting of operators (PISO) and skewness correction as pressure–velocity coupling, along with a pressure staggering option (PRESTO) scheme as pressure interpolation method and with second order accurate discretization was adopted. Convergence was enforced by reducing the residual of the continuity equation by 10^−6^ at every time step. For boundary conditions, the LAA wall was rigid with no-slip condition while flow velocity inlet (i.e., pulmonary veins) and outlet (mitral valve) with zero-pressure condition were set as flow conditions. For mitral valve outflow, a representative flow waveform with duration of 0.8 s at the mitral valve section was considered [25]. Afterwards, the inflow conditions were achieved by splitting the mitral outflow with a criterion based on proportionality of each pulmonary vein cross-sectional area on the basis of mass balance conservation. Each inlet and outflow were extended four times to ensure a fully developed flow at the entrance. To reduce the effect of transient flow, simulations were continued for three cardiac beats with the last cycle used for flow evaluation. The optimal solution was found for a time step of 0.02 s (i.e., 400 steps for each cardiac cycle). For comparison, a reference model using the undeformed LAA configuration was also developed.

## 3. Results

**Figure 2:**
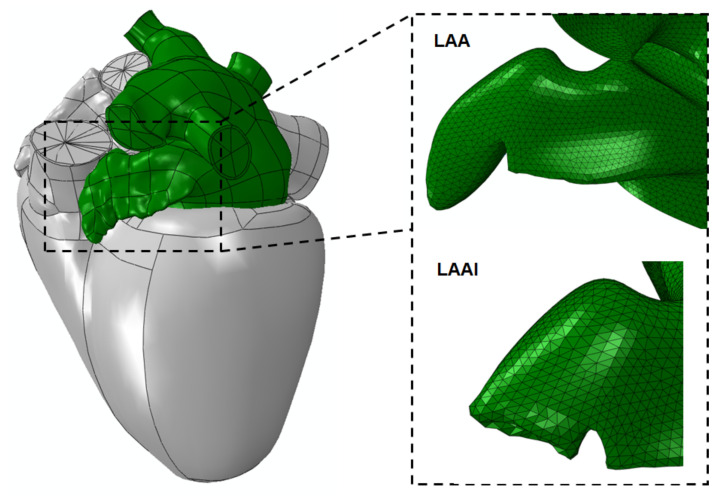
LA model (green) as extrapolated from the LHHM (grey); insertions show LAA mesh at undeformed and deformed configurations.

Figure 2 shows the left atrial model with the LAA mesh at both undeformed and deformed configurations. The inversion of LAA was successfully simulated upon the neck region of the appendage tissue wall.

**Figure 3:**
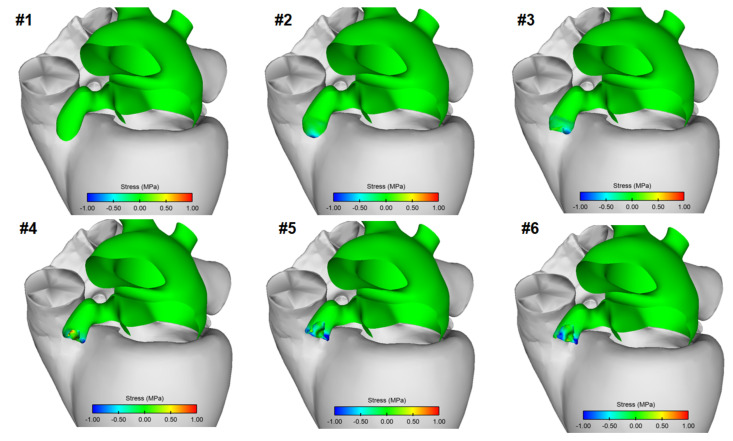
Color-coded stress distributions in the human left atrium during inversion of the LAA. Highest compressive (negative) stress (-1 MPa) is shown in dark blue; highest tensile (positive) stress (1 MPa) is shown in dark red. The progression of the procedure is from **#1** to **#6**. **#1**: Baseline, before any LAA inversion. **#2**: LAA is 20% inverted; compressive stress is seen near tip of LAA. **#3**: LAA is 40% inverted; compressive stress is seen over a larger portion of the LAA. **#4**: LAA is 60% inverted; compressive, as well as some tensile stress, is visible in LAA. **#5**: LAA is 80% inverted; only compressive stress is visible in LAA. **#6**: LAA is 100% inverted; only compressive stress is visible in LAA.

Figure 3 displays the map of the circumferential stress of LAA tissue wall at different steps of the inversion procedure. Compressive stress occurred in correspondence of the LAA tip as it was 20% inverted. Compressive, as well as some tensile, stresses were visible in the folded region of the LAA wall after simulation reached the 60% inversion. Only compressive stress was found in the LAA wall after the procedure was at 80% and 100% of the inversion. Local maxima of ~2.97 MPa compressive stresses were found at the end of the LAAI procedure.

**Figure 4:**
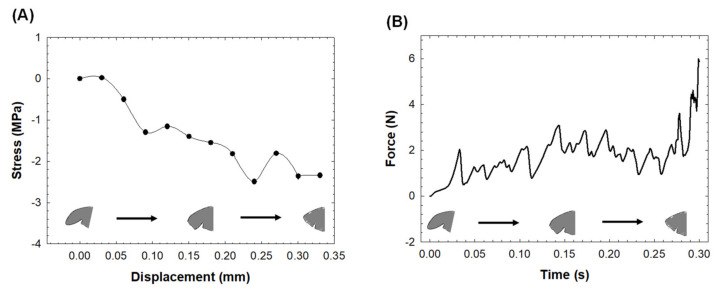
(**A**) Average stress at LAA tip as a function of displacement of the clamped nodes: (**B**) pulling force needed for LAAI as a function of simulation time.

Specifically, Figure 4A shows the stress profile as a function of the displacement of the LAA tip, and Figure 4B shows the pull force to invert the LAA. The stress at the end of simulation remains at the compressive state, with an average force to pull the LAA tip wall of 1.7 N for the whole simulation procedure.

**Figure 5:**
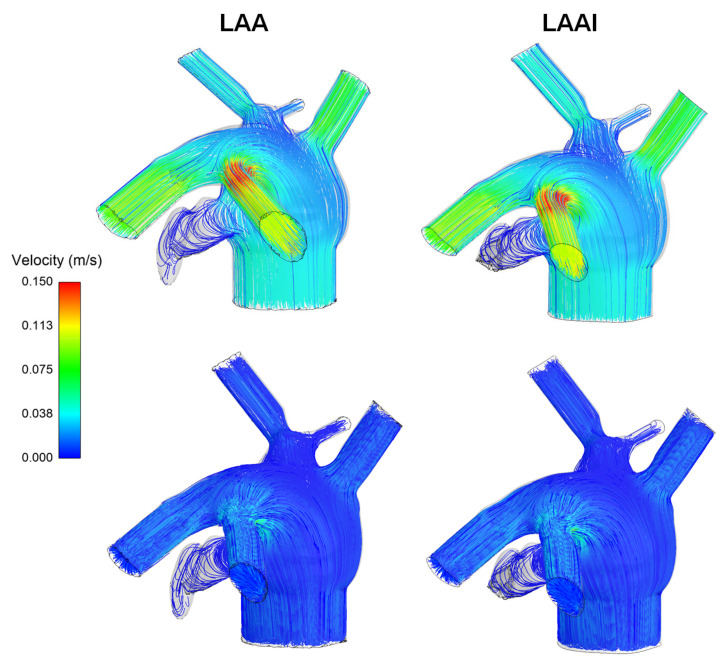
Flow velocity streamlines in the atrial chamber at peak systole of E-wave (**top** row) and diastole (**bottom** row) for both the non-inverted and inverted LAA.

Flow velocity at LAA was analyzed by streamlines at mitral valve flow peak of the E-wave and early diastole for both the non-inverted and LAAI models (Figure 5). At the peak velocity of E-wave, flow patterns were similar between the reference and LAAI models and were characterized by parallel flow streamlines with pronounced flow velocity at the branch of the pulmonary veins. At the early diastole, just after the deceleration phase of the A-wave, the flow velocity at the LAA was low with magnitude comparable to that of LA.

**Figure 6:**
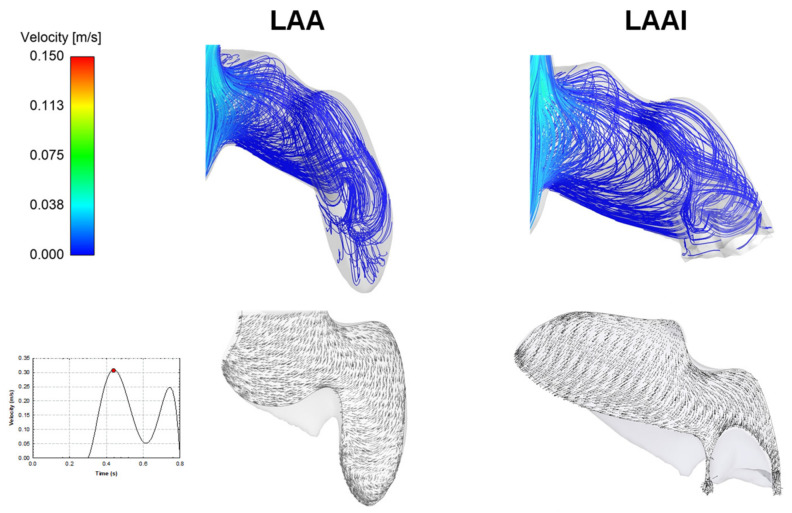
Flow velocity streamlines (top row) and velocity vector at a cross section for both the LAA and LAAI.

From a qualitative perspective, there was a minimal difference in the flow pattern of the LAAI model versus the non-inverted reference model (Figure 6). Specifically, the LAAI model was characterized by a minimally-nested helical flow pattern with low velocity magnitude near the tip of the inverted appendage.

**Figure 7:**
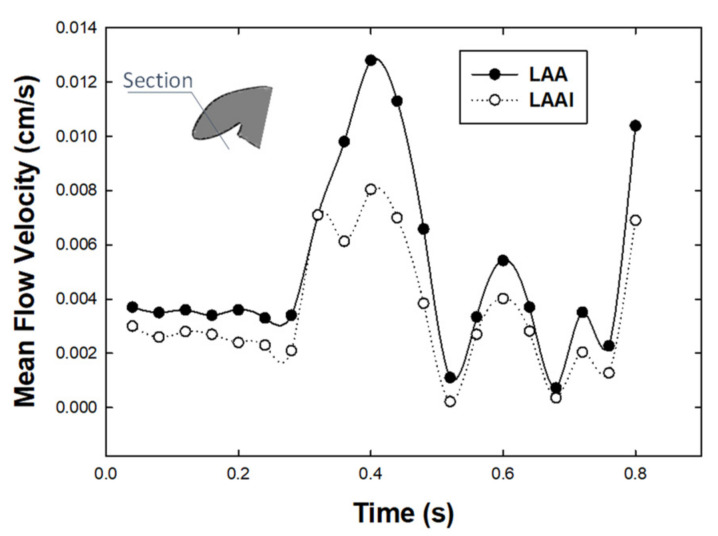
Mean flow velocity over one cardiac cycle at the tip of LAAI and in a cross-section of LAA (see inset).

Figure 7 highlighted that the mean flow velocity in a cross section at the LAAI tip over one cardiac cycle was slightly higher than that computed for the non-inverted reference model during diastole and the E-wave.

## 4. Discussion

Using realistic left atrial anatomy as extracted from the LHHM, the LAA tip was virtually clamped, and the retraction procedure was simulated to reveal insights into the biomechanics of the inverted LAA tissue wall (Figure 1 and Figure 2). The simulation confirmed the fundamental hypothesis of our study, suggesting a change in the stress distribution of the LAAI tissue wall from tensile to compressive (Figure 3) because of the inversion procedure. The circumferential stress of the LAAI tissue wall decreased upon 2.2 MPa at the end of inversion procedure, with the inversion process requiring a mean pull force of 1.7N (Figure 4). This compressive stress field can ultimately lead to resorption of the tissue as per reduced stress/resorption relation. Current LAA closure devices, whether epicardial or endocardial, must leave a device in the heart permanently, which may cause LAA perforation, migration, incomplete closure, new thrombus formation, and even thromboembolic events [26,27,28]. The concept of LAAI here proposed to promote resorption has not been previously reported and requires no permanent devices or implants (tissue glue or “stich” can be placed to ensure inversion configuration). We have here demonstrated the feasibility of such an approach by numerical simulation of LAAI in a realistic human model.

It has long been recognized that mechanics play a fundamental role in tissue growth and remodeling, especially in cardiac diseases. Mechanobiological control mechanisms in the myocardial wall tend to restore values of stress (or strain) toward preferred homeostatic values in response to diverse perturbations from normal conditions. For instance, hypertrophic growth of cardiomyocytes is the primary response by which the left ventricle reduces the stress on the myocardial wall imposed by pressure overload (e.g., hypertension) [29]. An increase in the tensile stress leads to intracellular signaling cascades that promote protein synthesis with consequent increases in the size and organization of cardiomyocytes and ultimately increasing the myocardium mass. Conversely, cardiac atrophy is a prevalent pathology associated with failed hearts after prolonged use of ventricular assist devices [30], resulting in an unloaded condition that causes tissue resorption. Based on these considerations, we expect that the compressive stress distribution generated on LAA wall by the inversion procedure could result in the resorption of the LAA tissue. Since the inverted state of the LAA offloads the stress (see Figure 3), we expect to resorb the apex (“blind end”) of the LAA and hence eliminate the dead space. This hypothesis requires validation in chronic experiments because, although changes in atrial cardiomyocytes may occur within hours, changes at the cardiac wall level occur over days to weeks or months. The distribution of stress changes in the LAAI model can be considered as the basis for the development of mathematical models of cardiac growth and remodeling. Such an approach would be especially useful in understanding and predicting the long-term biological response of LAAI and its mechanistic link with the stress level exerted on the LAA wall. The coupling of finite-element analysis with growth and remodeling, however, is complicated by the complexity of cardiac tissue.

From a hemodynamic perspective, the computational flow analysis revealed minimal differences in the flow patterns of the non-inverted LAA versus the LAAI (Figure 5 and Figure 6), which was characterized by slightly reduced flow velocities. In this context, computational flow analysis has been used to predict the hemodynamic disturbances of LAA and the risk of complications in the setting of treated and untreated AF. Using moving wall capability, simulations predicted well the risk of LAA thrombosis in a small cohort of patients and demonstrated that both wall kinetics and LAA shape contribute the development of blood stagnation to the LAA [31]. Computational flow analysis also demonstrated that not only complex LAA shapes have low velocities and vorticity indexes and consequently high risk of thrombogenic events, but even simple morphologies may have thrombogenic risk equal to, or even higher than, more complex auricles [32]. Among LAA phenotypes, the Windsock LAA shape is associated with a high risk of thrombosis as compared to that of Cauliflower morphology, according to computational estimations of blood washout [33] and clinical evidence [34]. Our findings from computational flow analysis corroborate the low blood flow velocity pattern in the LAA geometry, and this suggests the risk of thrombosis for the LAAI model given the low velocity, which appeared similar to that of the non-inverted LAA. It is evident, however, that the LAAI procedure remarkably reduced the area of blood stasis because of inverted tissue wall occupying the auricle, ultimately determining AF-related ischemic stroke by detachment of thrombus material. Given the association between LAA phenotype and function, further studies on different LAA shapes are needed to better understand the development of thrombus formation after simulation of the LAAI procedure.

There are several limitations in this numerical proof of concept study. First, for the sake of simplicity, the LAA tissue wall was assumed to be a passive and isotropic material with uniform thickness. During heart beating, contractile material force is initiated through changes in the electrical potential and depends on reference tension, the primary fiber stress ratio, the fiber-stretch velocity and the current cellular state. Second, knowledge of myofiber orientation is a crucial for model development even in a non-contracting myocardium because myocardial mechanical properties are significantly stiffer in the local myofiber direction than in a plane transverse to the myofiber direction. Our group has extensively studied and developed constitutive material law to account for the active material contraction and includes myofiber orientation as validated against in-vivo measured strain data [35,36]. Further studies will be undertaken to consider a more realistic constitutive behavior for the atrial chamber to refine predictions of LAAI biomechanics and reduce the impact of model assumptions. Third, in computational flow analyses, the rigid LA wall did not allow us to include the LA volume variation induced by the atrial contraction, likely resulting in a flow-rate change over the cardiac cycle. Fourth, no turbulence model was included, even though recent studies demonstrated the presence of a transitional flow in specific regions of the atrial chamber, but depending on patient anatomy. Indeed, The LAA morphology can play an important role in the development of turbulent flow conditions. In fact, the cauliflower-type LAA may lead to low blood flow velocity and vorticity. In future studies, the impact of turbulence on the resulting hemodynamic of LAAI will be therefore investigated. However, this study was carried out to assess the overall impact of the LAAI hemodynamic, rather than perfecting the model representation and fidelity. Most importantly, this study was not developed to test the resorption of the inverted appendage for which a stress-growth law modeling the resorption of the biological tissue subjected to stress reduction is required. Finally, the present study was developed only on a single LAA geometry. Thus, the simulation framework here proposed will be applied in a large patient cohort to validate the results of the current proof of concept study.

## 5. Conclusions

As a proof-of-concept, this study demonstrated the feasibility of the LAAI procedure for removal of the dead space of the appendage without leaving any device behind. Although further model improvements are needed, the simulation framework here proposed can be used not only to quantify the biomechanics and hemodynamic of LAAI but also to optimize LAAI procedure development towards translation in clinical practice.

## Data Availability

Not applicable.
